# Dr K. Bhaskaran (1924–2006)

**Published:** 2006

**Authors:** S. Kalyanasundaram

**Affiliations:** * Secretary General (RFS) and Chief Executive Officer, (RFS, Bangalore) Richmond Fellowship Society (RFS) of India, Bangalore, e-mail: sundarps@vsnl.com


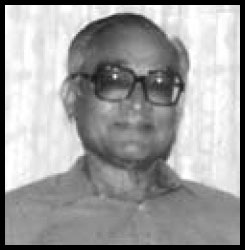


Dr K. Bhaskaran (1924–2006) obtained his MBBS degree from Madras University and Doctor of Medicine from Patna. He went on to complete his FRCP from Canada and later from London. He also got his Diploma in Psychiatry from McGill University and was made a Fellow of the Royal Australian and New Zealand College of Psychiatry. A bright student throughout his medical career, he held very senior positions in the government; some of them being:

- Member, Mental Health Expert Committee of the Indian Council of Medical Research (ICMR), 1963–74
- Director and Professor of Psychiatry, All India Institute of Mental Health (now National Institute of Mental Health and Neuro Sciences [NIMHANS]), Bangalore, March 1969–June 1969
- Member, Governing Body of the All India Institute of Mental Health (now NIMHANS), Bangalore, 1969–76
- Adviser, Mental Health to the Government of India (1971–75)
- Medical Superintendent, Hospital for Mental Diseases and Director, Post Graduate Centre (now Central Institute of Psychiatry), Ranchi, 1961–75
- Deputy Director-General of Health Services, New Delhi, 1975–76
- Served as WHO Consultant in Psychiatry
- Visiting Professor of Psychiatry, University of Pennsylvania, Philadelphia, Pa, USA, 1973–75

He also received the Sandoz Award (1968) and Dr Murthy Rao Oration Award (1992) of the Indian Psychiatric Society (IPS). He was President of the IPS during 1970–71. In fact, that was the time I first met Dr Bhaskaran in Madurai, at the Annual Conference of IPS. I was an intern having just completed my MBBS. I was most impressed by his scholarly look and the way he carried himself. He was erudite, was a good speaker and was impressed the way he was able carry his audience with him. I have since followed his career with admiration and respect and must admit that he was one of those who inspired me to take up Psychiatry as a profession.

His commitment to the profession and compassion to his patients was well known. He was a good teacher and even today some of his students, who themselves have held very high positions in both academia and practice, will vouch for that. Over the past 30 and more years I have known him, I had also become close to him and his family. His warmth and affection were infectious. One thing that impressed me was despite the fact that professionally I was very junior he would listen to me patiently and take my opinion in professional matters as if he was discussing with his peers. This aspect of humility has made a deep impact on me. He was a caring husband and a wonderful father to his two daughters, Gita and Nandini. The former took after her father and is a successful psychiatrist herself.

Dr Bhaskaran's academic pursuit can be gauged from the fact that he had contributed more than 110 original articles and chapters in textbooks that were published both in national and international journals. His areas of interest included rehabilitation of patients with schizophrenia with special reference to home care, undergraduate and postgraduate psychiatric education, and psychotherapy and meditation from mental health perspective.

The field of Psychiatry in India will definitely miss this personality who exhibited benevolence for all and malice towards none. The psychiatric community has lost one of its illustrious sons.

